# Tomato fruit quality is more strongly affected by scion type and planting season than by rootstock type

**DOI:** 10.3389/fpls.2022.948556

**Published:** 2022-12-15

**Authors:** Tian Gong, Jeffrey K. Brecht, Samuel F. Hutton, Karen E. Koch, Xin Zhao

**Affiliations:** ^1^Horticultural Sciences Department, University of Florida, Gainesville, FL, United States; ^2^Gulf Coast Research and Education Center, University of Florida, Wimauma, FL, United States

**Keywords:** rootstock–scion synergy, generative rootstock, vegetative rootstock, grape tomato, beefsteak tomato, fruit physical profile, fruit composition, rootstock vigor

## Abstract

Previous studies of tomato rootstock effects on fruit quality have yielded mixed results, and few attempts have been made to systematically examine the association between rootstock characteristics and tomato fruit quality. In this study, grape tomato (‘BHN 1022’) and beefsteak tomato (‘Skyway’) were grafted onto four rootstocks [‘Estamino’ (vigorous and “generative”), ‘DR0141TX’ (vigorous and “vegetative”), ‘RST-04-106-T’ (uncharacterized), and ‘SHIELD RZ F1 (61–802)’ (mid-vigor, uncharacterized)] and compared to non-grafted scion controls for two growing seasons (Spring and Fall in Florida) in organically managed high tunnels. In both seasons and for both scions, the two vigorous rootstocks, regardless of their designation as “vegetative” (‘DR0141TX’) or “generative” (‘Estamino’), exhibited negative impacts on dry matter content, soluble solids content (SSC), SSC/titratable acidity (TA), lycopene, and ascorbic acid contents. Similar effects on fruit dry matter content and SSC were also observed with the ‘RST-04-106-T’ rootstock, although little to no change was seen with grafting onto ‘SHIELD RZ F1 (61–802)’. Further studies are needed to elucidate the impact of rootstock vigor on tomato volatile profiles and consumer sensory acceptability in order to better determine whether any of the documented effects are of practical importance. On the other hand, the evident effects of scion cultivar and planting season on fruit quality were observed in most of the measurements. The scion by rootstock interaction affected fruit length, firmness, pH, and total phenolic content, while the planting season by rootstock interaction impacted fruit firmness, pH, total antioxidant capacity, and ascorbic acid and lycopene contents. The multivariate separation pattern of planting season, scion, and rootstock treatments as revealed by the canonical discriminant analysis further indicated that the influence of scion cultivar and planting season on tomato fruit quality could be much more pronounced than the rootstock effects. The fruit color (C* and *H*°), length and width, SSC, pH, total antioxidant capacity, ascorbic acid, and lycopene contents were the main attributes distinguishing different scion-planting season groups.

## Introduction

Tomato (*Solanum lycopersicon*) is widely grown around the world. In 2021, the production of tomato in the United States exceeded 10.5 million tonnes (USDA, 2022). Tomato fruit contains different classes of antioxidants such as carotenoids, ascorbic acid, phenolics, and tocopherols. Because of its high *per capita* consumption, tomato contributes considerably to the dietary intake of antioxidant phytochemicals (Nicoletto et al., 2013). Tomato grafting has attracted increasing interest, especially from organic producers. This interest promotes the development of new rootstocks for tomato grafting. Commercial tomato rootstocks have been mainly categorized by their resistance to soil-borne diseases and growth vigor by commercial suppliers. The rootstock vigor has been suggested to impact fruit quality (Krumbein and Schwarz, 2013); however, systematic research is scarce with respect to understanding the relation between tomato rootstock vigor and fruit compositional properties.

Although rootstock impacts on tomato fruit quality have been examined in many studies, using rootstocks with distinct disease resistance packages and other growth characteristics together with the various possible rootstock–scion combinations has led to mixed results. Under greenhouse growth conditions, neutral or negative effects of rootstock on tomato fruit firmness, dry matter content, pH, soluble solids content (SSC), total soluble sugars, titratable acidity (TA) (also positive effects of rootstock observed), SSC/TA, ascorbic acid, lycopene (also positive effects of rootstock observed), and *β*-carotene contents have been reported (Turhan et al., 2011; Abu Glion et al., 2019; Liu et al., 2019; Sun et al., 2021). In addition, it was found that rootstock did not affect soluble sugars (Sun et al., 2021) in greenhouse production systems. Under high tunnel growing conditions, Nicoletto et al. (2013) revealed that rootstocks did not affect beefsteak tomato fruit pH and TA but decreased SSC, antioxidant activity, total phenols, and ascorbic acid content relative to the self-grafted control regardless of ripening conditions. The production system seems to be another driving factor for tomato fruit quality of grafted plants. Interestingly, when using the same beefsteak tomato scion and producing under high tunnel conditions, Lang et al. (2020) found that fruit firmness, SSC, pH, TA, and SSC/TA varied with rootstocks and years, whereas Meyer et al. (2019) reported an absence of rootstock impacts on fruit SSC. In addition, the production system by rootstock interaction effect on tomato fruit quality has also been reported by Khah et al. (2006).

Some researchers deliberately included rootstocks with contrasting vigor or with different genetic backgrounds in their studies to examine the effects of these characteristics on tomato fruit quality. In a greenhouse study, Mauro et al. (2020) evaluated the fruit quality of a medium-sized, elongated tomato after grafting onto rootstocks with different vigor and found that fruit dry matter content was similar among rootstocks of different vigor, and fruit firmness, SSC, TA, SSC/TA, *β*-carotene content, and ascorbic acid content variations did not show a clear pattern as rootstock vigor increased, while fruit lycopene content decreased with increased rootstock vigor. In a 2-year open-field study carried out in Spring season, no differences were found in SSC, pH, TA, SSC/TA, and ascorbic acid content among non-grafted beefsteak tomato and those grafted with interspecific rootstock or intraspecific rootstock (Barrett et al., 2012). The lack of impacts on these fruit quality attributes from interspecific rootstocks was also observed by Djidonou et al. (2016), except for reduced ascorbic acid content. In addition to growth of vigor, the so-called “generative” or “vegetative” rootstock effects on tomato scions have been claimed recently by some commercial suppliers to describe certain tomato rootstock cultivars. Lopez-Marin et al. (2017) suggested that generative rootstocks tended to put more energy into reproductive tissue than into vegetative tissue. Such different patterns of photosynthate partitioning could potentially result in differential effects on tomato fruit quality.

As tomato fruit quality can be markedly impacted by environmental conditions associated with different growing seasons (Anza et al., 2006; Raffo et al., 2006), the rootstock effects on fruit quality may also potentially vary with growing seasons. Temperature is one of the key drivers of environmental differences between growing seasons and has been shown to significantly affect tomato fruit quality (Beckles, 2012). Much attention has been paid to rootstock impacts on tomato plant growth or yields under different temperatures (Rivero et al., 2003; Ntatsi et al., 2014; Suchoff et al., 2018), but very little research has been done examining the influence of growing temperature on grafted tomato fruit quality. Riga (2015) reported that under winter greenhouse production with low temperature (daily average temperature varied between 15 and 20°C) and light intensity, tomato fruit firmness, dry matter content, SSC, and SSC/TA were rootstock cultivar-dependent. However, the studies conducted under greenhouse conditions or with preset constant temperatures within a predefined time frame, may not fully represent seasonal-related fruit quality variations in open fields or under high tunnels. In north central Florida, tomato planting under high tunnel conditions can be conducted in two distinct production seasons in the Spring and in the Fall, which enables the characterization of growing season impacts on grafted tomato fruit quality.

In most tomato grafting research, fruit quality has been evaluated when scions of similar types were grafted with rootstocks possessing different characteristics, whereas no direct comparison has been made between different tomato types when grafted with the same rootstocks. The objectives of this study of grafted tomato production in organically managed high tunnels were to examine: (1) the impacts of rootstocks with different vigor and other characteristics on the fruit quality attributes of grafted beefsteak and grape tomatoes; and (2) the effects of planting season on the quality attributes of those grafted tomatoes.

## Materials and methods

### Tomato production

Experiments were conducted in the Spring (hereafter referred to as Spring planting) and Fall (hereafter referred to as Fall planting) seasons of 2020 at the University of Florida Plant Science Research and Education Unit in Citra, FL. In both seasons, determinate ‘BHN 1022’ grape tomato (BNHSeed, Immokalee, FL, United States) and ‘Skyway’ beefsteak tomato (Johnny’s Selected Seeds, Winslow, ME, United States) were grafted onto the following tomato rootstocks: ‘DR0141TX’ (vegetative) (De Ruiter Seeds, Bergschenhoek, Netherlands), ‘Estamino’ (generative) (Vitalis Organic Seed, Salinas, CA, United States), ‘RST-04-106-T’ (uncharacterized) (NE Seed, East Hartford, CT, United States), and ‘SHIELD RZ F1 (61–802)’ (referred to as ‘Shield’ thereafter; uncharacterized) (Rijk Zwaan, De Lier, Netherlands). Based on our previous rootstock research (Gong, 2022), among these four rootstocks, ‘DR0141TX’ and ‘Estamino’ (*S. lycopersicum* × *S. habrochaites*) (Ministry of Agricultural, Food and Forestry Policies, 2021) are of high vigor, while ‘Shield’ (*S. lycopersicum*) is of low vigor, and ‘RST-04-106-T’ is of intermediate vigor between ‘DR0141TX’/‘Estamino’ and ‘Shield’.

A split-plot design with four replications (four high tunnels) was used with scion type (beefsteak and grape tomato cultivars) being the whole plot factor and different rootstock treatments together with non-grafted scion controls in the subplots. There were eight plants in each subplot. Rootstock and scion seedling production and grafting were performed as described in Gong (2022). Briefly, tomato rootstocks and scions were seeded on 24 and 28 December 2019, respectively, for Spring planting, while rootstocks and scions were seeded on 1 and 5 August 2020, respectively, for Fall planting. Splice grafting (Lee and Oda, 2002) was conducted when plants had three to four true leaves on 21 January 2020 and 7 September 2020 for Spring and Fall plantings, respectively. Grafted tomato plants were transplanted into raised beds with plastic mulch and drip irrigation on 14 February 2020 and 24 September 2020 for the Spring and Fall plantings, respectively, for production in unheated, passively ventilated high tunnels (2.76 m high, 4.27 m wide, and 30.48 m long) on certified organic land.

Yard waste–based compost (Watson C&D, Gainesville, FL, United States) was applied at 22.4 and 16.8 t/ha to the tomato planting beds for Spring and Fall plantings, respectively. The Nature Safe 10-2-8 organic fertilizer (Darling Ingredients Inc., Irving, TX, United States) was applied to the planting beds at a rate of 112 kg/ha N for both planting seasons. The Aqua Power 5-1-1 liquid fish fertilizer (JH Biotech, Inc., Ventura, CA, United States) and Big K 0-0-50 potassium sulfate (JH Biotech, Inc.) were mixed and injected at the rates of 11.8–34.0 kg N and 9.8–28.2 kg K per ha per week during the production season.

The whole season average, maximum, and minimum air temperatures were 22.7, 37.5, and 2.4°C for Spring planting and 18.6, 35.2, and 1.5°C for Fall planting, respectively. In general, the soil-borne disease and nematode pressure were low in Spring and Fall plantings except that the non-grafted ‘BHN 1022’ was affected by a medium level of root-knot nematode infestation (nematode galling index rating of 4.1 based on a 0–10 scale) in Fall planting.

### Fruit sampling and processing

Tomato harvests began on 20 April 2020 for both beefsteak and grape tomatoes in Spring planting. In Fall planting, the first grape tomato harvest was on 29 November 2020, but the beefsteak harvest was delayed to 28 December 2020. Fruit in both trials were harvested twice per week. Grape tomatoes were harvested when they reached a uniform, complete red color with a tinge of orange, while beefsteak tomatoes were harvested at the breaker stage (when there was a definite break in color from green to tannish-yellow, pink, or red on not more than 10% of the surface). Fruit were sampled for quality assessment during the peak harvest period. In Spring planting, sampling was on 14 May 2020 and 21 May 2020 for grape tomato and beefsteak tomato, respectively. In Fall planting, sampling was on 8 January 2021 and 2 February 2021 for grape tomato and beefsteak tomato, respectively. Grape tomato fruit were processed within 1 day while beefsteak tomato fruit were ripened to full ripe (100% red color) at 20°C and 45% relative humidity before processing.

For grape tomato, about 800 g marketable fruit (approximately 70 fruit) with uniform size and color were chosen from each subplot. Fruit were not washed in Spring planting, but since an organic pesticide product had to be used for pest control a few days before fruit sampling in Fall planting, those fruit were rinsed with the diluted sodium hypochlorite solution (210 μL/L) followed by tap water and DI water and dried with paper towels before processing. For the treatment in each subplot, ten fruits were randomly picked for the measurements of length, width, and color; a separate set of 10 grape tomatoes were used for firmness assessment. Around 600 g of fruit per subplot were further processed for compositional analysis.

For beefsteak tomato, 10–14 marketable fruits from each subplot were randomly selected for fruit quality assessments. In both planting seasons, they were rinsed using the same method as for grape tomato. When the increase of a* value measured near the blossom end with a chromameter ceased increasing from one day to the next, the tomatoes were considered to be fully ripe (Tulio et al., 2009). A set of six representative fully ripe fruit from each subplot were then processed for quality assessments. Following fruit size, color, and firmness measurements, a fruit sample of about 600 g from each subplot was processed for chemical profile analysis.

### Fruit color, size, firmness, and dry matter content measurements

Fruit size was measured to the nearest 0.1 mm with a digital caliper. Fruit length was measured from the stem scar to the blossom end, and fruit width was measured at the largest part at the equator. Fruit color was measured at one point at the equator for grape tomato and at two points on opposite sides at the equator for beefsteak tomato using a chromameter with an 8-mm aperture (CR-400; Minolta, Tokyo, Japan). The CIELAB system (L*, a*, b*) was used and numerical values of a* and b* were converted into hue angle [*H*° = tan^–1^ (b*/a*)] and chroma ($C^{*}=\sqrt{a^{*2}+b^{*2}})$. Hue represents the shade of color, while chroma represents the purity of a given hue (Loayza et al., 2021).

Firmness of beefsteak tomatoes was measured at the blossom end using a handheld penetrometer (Fruit Tester; Wagner Instruments, Greenwich, CT, United States) with an 11.3-mm diameter probe. A thin layer of skin at the blossom end was peeled from the beefsteak tomato, and the probe was centered on the blossom end before measurement. Firmness was expressed as the maximum resistance force produced during penetration to a depth of 8.33 mm. Firmness of grape tomato was similarly measured at the equator using the same firmness tester with an 8-mm diameter probe (penetrating to 7.3 mm deep) but with the skin on.

Whole grape tomato fruit and quartered beefsteak tomato fruit samples (600 g) were homogenized with a commercial bar blender (HBB908, Hamilton Beach Brands, Inc., Glen Allen, VA, United States) under yellow light. After blending, about 100 g of each homogenized sample were poured into an aluminum bowl for dry matter content determination. The fresh weight of each sample was recorded and then the samples were dried at 65°C to constant weight with the dry weight recorded. Dry matter content was calculated as the ratio of dry weight to fresh weight and expressed as a percentage. Additional homogenized tissue was stored in zipper-lock plastic freezer bags at -30°C until further analysis.

### Soluble solids content, titratable acidity, and pH

Before measuring SSC, TA, and pH, homogenized fruit samples were thawed at 4°C and 35 g of each fruit sample were put in a 50-ml centrifuge tube and centrifuged (Sorvall Lynx 4000 Centrifuge; Thermo Fisher Scientific, Waltham, MA, United States) at 12,000 × *g* for 20 min at 4°C. The resultant supernatant was filtered through two-layer cheesecloth and used for the measurements. A few drops of the filtered supernatant were placed on the prism of a digital refractometer (model r2i300; Reichert Analytical Instruments, Depew, NY, United States) to measure SSC in Brix. A 3.0-ml aliquot of the supernatant was mixed with 50 mL DI water for measuring TA and pH, using a 905 Titrando automatic titration system (Metrohm USA Inc., Riverview, FL, United States) with TA measurements expressed as percent citric acid.

### Analysis of total antioxidant capacity

Total antioxidant capacity of the homogenized tomato samples was determined based on the ferric reducing antioxidant power (FRAP) assay according to Benzie and Strain (1996) and Frey (2018) with modifications. Briefly, 0.5-ml filtered supernatant was diluted with 9.5 mL DI water, and triplicate 50 μL aliquots of the diluted sample were used for this assessment. The absorbance was read at 593 nm with a microplate reader (Synergy HTX, BioTek Instruments, Inc., Winooski, VT, United States). Total antioxidant capacity was calculated as mg of Trolox equivalent per 100 g FW.

### Analysis of antioxidant compounds

Contents of total carotenoids, lycopene, *β*-carotene, ascorbic acid, and total phenolics were measured in both planting seasons. Tomato total carotenoid content of the homogenized tomato samples was analyzed according to Lee and Castle (2001) and Frey (2018). A 2.0-g aliquot of homogenized tomato tissue was used, and absorbance was read at 470 nm, using hexane solvent as a blank. Total carotenoid content was calculated as mg of *β*-carotene equivalent (*β*CE) per 100 g FW. Tomato lycopene and *β*-carotene contents were analyzed according to Nagata and Yamashita (1992) and Frey (2018). A 1.0-g aliquot of homogenized tomato tissue was used, and absorbance was measured at 453, 505, 645, and 663 nm, using hexane solvent as a blank. Lycopene and *β*-carotene contents were calculated as μg per 100 g FW.

For ascorbic acid measurement, immediately after blending the fresh tomato samples, 2.0 g of each homogenized sample were put in a 50-mL screw cap centrifuge tube (Thermo Fisher Scientific, Waltham, MA, United States) and 20 mL of an acid mixture (containing 6% metaphosphoric acid in 2 N acetic acid) was added to prevent ascorbic acid degradation. All samples were frozen at -30°C until further analysis. Tomato ascorbic acid content was analyzed according to a modified AOAC method 967.21 according to AOAC International (1995) and Frey (2018). An ascorbic acid standard curve was prepared with 0.5, 1.0, 2.0, 3.0, 4.0, and 5.0 μg ascorbic acid per mL of 6% metaphosphoric acid in 2 N acetic acid. Absorbance was read at 540 nm. Ascorbic acid content was calculated as mg of ascorbic acid per 100 g fresh weight (FW).

Tomato total phenolic content was analyzed by hydrophilic extraction and the Folin–Ciocalteu reaction according to Singleton and Rossi (1965) and Frey (2018) using gallic acid as the standard in a calibration curve from 0.02 to 0.10 mg/mL at 0.02 mg/mL intervals. A 0.5-mL aliquot of filtered supernatant was used, and the absorbance was measured at 765 nm. Total phenolic content was expressed as mg of gallic acid equivalent (GAE) per 100 g FW.

### Statistical analysis

The experimental layout in the high tunnels followed a split-plot design. In order to examine seasonal effects on the fruit quality of grafted plants, planting season was added to the model, making the experiment a split-split-plot design. Planting season was the whole plot factor, and scion cultivar was the subplot factor nested within planting season, while rootstock was the sub-subplot factor nested within the scion cultivar. Fruit quality data were analyzed using the GLIMMIX procedure of SAS (version 9.4; SAS Institute, Cary, NC, United States) following a generalized linear mixed model. Square root transformation was used for some data to meet the model assumptions, but the results were presented using the original data. Fisher’s least significant difference (LSD) test at *p* ≤ 0.05 was conducted for multiple comparisons of different measurements among treatments.

Canonical discriminant analysis along with the forward stepwise variable selection method based on *p* ≤ 0.05 was used in JMP Pro 15 (SAS Institute, Cary, NC, United States) to separate treatments based on the fruit quality attributes [fruit length, width, fruit color (L*, C*, *H*°), fruit firmness, dry matter content, pH, SSC, TA, SSC/TA, total antioxidant capacity, total carotenoid, lycopene, *β*-carotene, ascorbic acid, and total phenolic contents].

## Results

### Fruit color

No rootstock impacts were detected for L*, but the L* value was affected by planting season, scion, and planting season × scion interactions (Table 1). The L* values were higher (i.e., fruit skin color lighter) for both tomato scions in Spring planting than in Fall planting with a greater degree of increase in ‘BHN 1022’. The two scion cultivars showed similar L* values in the Spring, whereas ‘Skyway’ fruit had a higher L* value than ‘BHN 1022’ in the Fall (Table 2). Fruit C* was affected by planting season, rootstock, scion, and the three-way interactions among them (Table 1). For ‘BHN 1022’ grape tomato, rootstock effects were only detected in Fall planting as all rootstocks except the least vigorous rootstock ‘Shield’ decreased C* compared with the non-grafted control, indicating the colors of the fruit of vigorous rootstocks (‘DR0141TX’ and ‘Estamino’) and mid-vigor rootstock ‘RST-04-106-T’ were not as pure as that of the non-grafted control (Table 3). In contrast, for ‘Skyway’ beefsteak tomato, rootstock impacts were detected in Spring planting but not in Fall planting. In Spring planting, ‘DR0141TX’ treatments had lower C* (i.e., less pure color) relative to the fruit of the non-grafted control, while the other rootstock treatments were similar to the non-grafted control. In addition, fruit of the scion grafted onto ‘Shield’ had higher C* than those onto the two vigorous rootstocks ‘DR0141TX’ and ‘Estamino’. For all rootstock–scion combinations, C* values were higher in Spring than in Fall but with different degrees. The *H*° values were affected by the planting season, rootstock, and the two-way interactions between planting season and scion (Table 1). Grafting with ‘DR0141TX’ or ‘Estamino’ increased fruit *H*° compared with ‘Shield’ and the non-grafted controls by around 1 unit, while ‘RST-04-106-T’ were similar to all treatments (Table 2). For both scions, *H*° values were greater in Fall planting than in Spring planting with the increase being more prominent for the beefsteak tomato ‘Skyway’, indicating fruits were redder in the Spring than in the Fall. In addition, in Spring planting, ‘BHN 1022’ fruit showed higher *H*° values than ‘Skyway’, while this difference was absent in Fall.

**TABLE 1:** Analysis of variance of the effects of planting season (Spring and Fall), rootstock (‘DR0141TX’, ‘Estamino’, ‘RST-04-106-T’, ‘Shield’, and the non-grafted scion controls), and scion (grape tomato ‘BHN 1022’ and beefsteak tomato ‘Skyway’) on tomato fruit C* (chroma), L* (lightness), and *H*° (hue angle) values, length, width, firmness, and dry matter content.

Effect	L*	*C* *	*H*°	Fruit length	Fruit width	Firmness	Dry matter content
P	***	***	***	***	***	**	*
R	NS	**	**	***	***	***	***
S	***	***	NS	***	***	*	***
P × R	NS	NS	NS	NS	NS	*	NS
P × S	***	NS	**	***	***	***	*
S × R	NS	NS	NS	*	NS	*	NS
P × S × R	NS	*	NS	NS	NS	NS	NS

P = planting season; R = rootstock; S = scion; P × R = planting season × rootstock interaction; P × S = planting season × scion interaction; S × R = scion × rootstock interaction; P × S × R = planting season × scion × rootstock interaction. NS, *, **, *** Non-significant or significant at *p* at 0.05, 0.01, or 0.001, respectively.

**TABLE 2:** Fruit L* (lightness) and *H*° (hue angle) values of fruit from grafted beefsteak tomato ‘Skyway’ and grape tomato ‘BHN 1022’ in Spring and Fall plantings.

Treatment	L*		*H*°	
	BHN 1022	Skyway	BHN 1022	Skyway
**Planting season**				
Spring	40.82 ± 0.18 Aa	42.43 ± 0.18 Aa	44.17 ± 0.48 Ab	42.04 ± 0.48 Bb
Fall	37.52 ± 0.18 Bb	41.66 ± 0.19 Ab	46.62 ± 0.48 Aa	47.61 ± 0.48 Aa

**Rootstock**				
DR0141TX	40.69 ± 0.18		45.82 ± 0.37 a	
Estamino	40.79 ± 0.18		45.65 ± 0.37 a	
RST-04-106-T	40.47 ± 0.19		45.14 ± 0.38 ab	
Shield	40.57 ± 0.18		44.27 ± 0.37 b	
Non-grafted	40.52 ± 0.18		44.67 ± 0.37 b	

DR0141TX, tomato scions grafted onto rootstock ‘DR0141TX’; Estamino, tomato scions grafted onto rootstock ‘Estamino’; RST-04-106-T, tomato scions grafted onto rootstock ‘RST-04-106-T’; Shield, tomato scions grafted onto ‘Shield’; Non-grafted, non-grafted tomato scion controls. Mean ± SE (standard error); Means followed by the same uppercase letter within a row, and means followed by the same lowercase letter within a column are not significantly different at *p* ≤ 0.05 according to Fisher’s LSD test.

**TABLE 3:** Fruit C* (chroma) values of fruit from grafted beefsteak tomato ‘Skyway’ and grape tomato ‘BHN 1022’ in Spring and Fall plantings.

Scion	Rootstock	Spring planting	Fall planting
BHN 1022	DR0141TX	38.21 ± 0.44*Aa*	29.18 ± 0.44*Bc*
	Estamino	37.87 ± 0.44*Aa*	30.31 ± 0.44*Bbc*
	RST-04-106-T	37.22 ± 0.44*Aa*	29.83 ± 0.44*Bbc*
	Shield	38.10 ± 0.44*Aa*	30.76 ± 0.44*Bab*
	Non-grafted	37.48 ± 0.44*Aa*	31.57 ± 0.44*Ba*

Skyway	DR0141TX	40.29 ± 0.44*Ac*	34.23 ± 0.44*Ba*
	Estamino	40.84 ± 0.44*Abc*	33.28 ± 0.44*Ba*
	RST-04-106-T	41.94 ± 0.44*Aab*	33.88 ± 0.51*Ba*
	Shield	42.19 ± 0.44*Aa*	34.84 ± 0.44*Ba*
	Non-grafted	41.49 ± 0.44*Aab*	34.71 ± 0.44*Ba*

DR0141TX, tomato scions grafted onto rootstock ‘DR0141TX’; Estamino, tomato scions grafted onto rootstock ‘Estamino’; RST-04-106-T, tomato scions grafted onto rootstock ‘RST-04-106-T’; Shield, tomato scions grafted onto ‘Shield’; Non-grafted, non-grafted tomato scion controls. Mean ± SE (standard error); Means followed by the same uppercase letter within a row, and means followed by the same lowercase letter within a column are not significantly different at *p* ≤ 0.05 according to Fisher’s LSD test.

### Fruit size, firmness, and dry matter content

Both fruit length and width were affected by planting season, scion, and rootstock as well as planting season by scion interactions. Fruit length was also affected by the interaction between scion and rootstock (Table 1). For the grape tomato ‘BHN 1022’, fruit length was similar among all rootstock treatments (Table 4). For the beefsteak tomato ‘Skyway’, fruit from scion grafted with the vigorous rootstocks (‘DR0141TX’ and ‘Estamino’) had greater length than the non-grafted control that was similar to the mid-vigor rootstock ‘RST-04-106-T’ and the least vigorous rootstock ‘Shield’. In addition, ‘DR0141TX’ rootstock resulted in greater fruit length than ‘RST-04-106-T’ (by 4.7%) and ‘Shield’ (by 9.5%) while ‘Estamino’ resulted in greater in fruit length than ‘Shield’ (by 7.8%). As expected, fruit length was greater for the beefsteak tomato scion ‘Skyway’ than for the grape tomato scion ‘BHN 1022’, while the differences appeared to be greater when grafted with ‘DR0141TX’, ‘Estamino’, or ‘RST-04-106-T’. Fruit width was increased when scion cultivar was grafted with ‘DR0141TX’, ‘Estamino’, or ‘RST-04-106-T’ compared with ‘Shield’ and the non-grafted controls by 4% on average, with the latter two being similar. In terms of planting season effects on fruit length and width, it was found that for ‘BHN 1022’, fruit from the Fall planting were 7.5% longer than those from the Spring planting. However, the fruit length of ‘Skyway’ in Fall planting was 19.0% less relative to the Spring planting. Planting season did not affect the width of fruit of ‘BHN 1022’, whereas Fall planting decreased the width of ‘Skyway’ fruit by 17.4% compared with Spring planting.

**TABLE 4:** Fruit length, width, and dry matter content of fruit from grafted beefsteak tomato ‘Skyway’ and grape tomato ‘BHN 1022’ in Spring and Fall plantings.

Treatment	Length (mm)		Width (mm)		Dry matter content (%)	
	BHN 1022	Skyway	BHN 1022	Skyway	BHN 1022	Skyway
**Planting season**						
Spring	33.1 ± 0.6 Bb	95.3 ± 1.0 Aa	24.7 ± 0.4 Ba	76.5 ± 0.8 Aa	6.46 ± 0.15 Ab	5.01 ± 0.13 Ba
Fall	35.6 ± 0.6 Ba	80.1 ± 0.9 Ab	25.3 ± 0.4 Ba	65.1 ± 0.7 Ab	7.13 ± 0.16 Aa	5.03 ± 0.13 Ba

**Rootstock**						
DR0141TX	35.2 ± 0.7 Ba	91.4 ± 1.1 Aa	45.8 ± 0.5 a		5.62 ± 0.09 b	
Estamino	34.7 ± 0.7 Ba	90.0 ± 1.1 Aab	45.9 ± 0.5 a		5.62 ± 0.09 b	
RST-04-106-T	34.5 ± 0.7 Ba	87.3 ± 1.2 Abc	45.2 ± 0.5 a		5.81 ± 0.10 b	
Shield	34.0 ± 0.7 Ba	83.5 ± 1.1 Ad	43.7 ± 0.5 b		6.14 ± 0.09 a	
Non-grafted	33.3 ± 0.7 Ba	85.5 ± 1.1 Acd	44.0 ± 0.5 b		6.18 ± 0.09 a	

DR0141TX, tomato scions grafted onto rootstock ‘DR0141TX’; Estamino, tomato scions grafted onto rootstock ‘Estamino’; RST-04-106-T, tomato scions grafted onto rootstock ‘RST-04-106-T’; Shield, tomato scions grafted onto ‘Shield’; Non-grafted, non-grafted tomato scion controls. Mean ± SE (standard error); Means followed by the same uppercase letter within a row, and means followed by the same lowercase letter within a column are not significantly different at *p* ≤ 0.05 according to Fisher’s LSD test.

**FIGURE 1:**
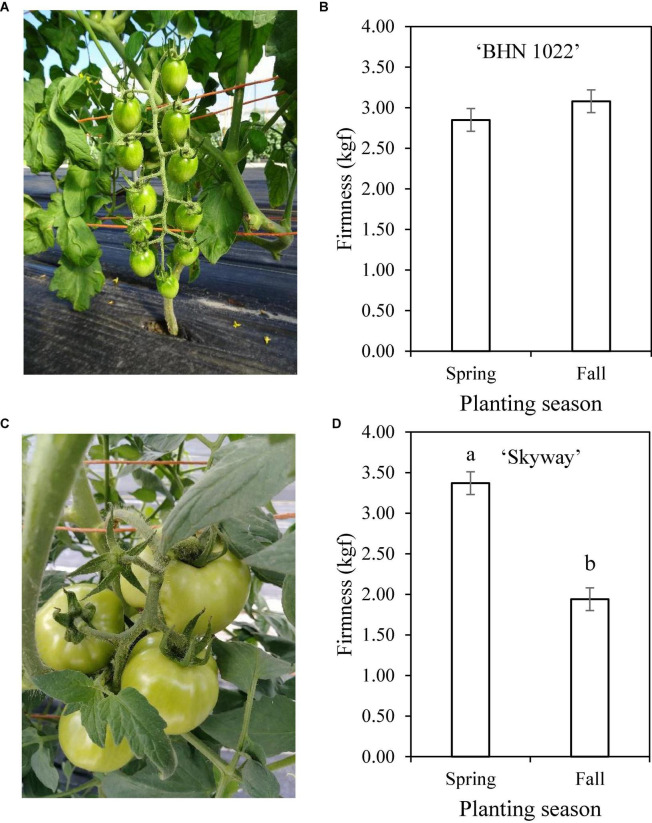
Tomato fruit firmness as affected by scion × planting season interaction. **(A)** ‘BHN 1022’ grape tomato scion. **(B)** Fruit firmness of ‘BHN 1022’. **(C)** ‘Skyway’ beefsteak tomato scion. **(D)** Fruit firmness of ‘Skyway’. Error bars represent standard errors. Means followed by the same letter are not significantly different at *p* ≤ 0.05 according to Fisher’s LSD test.

Planting season, rootstock, scion, and two-way interactions among them were detected for fruit firmness (Table 1). For the grape tomato ‘BHN 1022’, fruit firmness was similar among all treatments (Table 5). For the beefsteak tomato ‘Skyway’, grafting with vigorous rootstocks (‘DR0141TX’ and ‘Estamino’) decreased fruit firmness by 19.3% on average relative to the non-grafted control, and these two treatments also had lower firmness compared with the mid-vigor rootstock ‘RST-04-106-T’. When grafted with ‘DR0141TX’ or ‘Estamino’, ‘BHN 1022’ fruits were firmer than those of ‘Skyway’, while for the remaining rootstock treatments and the non-grafted plants, ‘BHN 1022’ fruits were similar to ‘Skyway’ fruit. In Spring planting, grafting with ‘DR0141TX’ or ‘Estamino’ decreased fruit firmness relative to ‘RST-04-106-T’, ‘Shield’, and the non-grafted controls for both scions by 22.0%, while in Fall planting, no differences in rootstock effects were detected. When grafting with ‘RST-04-106-T’ or ‘Shield’ or non-grafted, plants in Spring planting had firmer fruit than in Fall planting. Furthermore, fruit firmness of the grape tomato ‘BHN 1022’ (Figure 1A) was not impacted by planting season (Figure 1B), whereas Fall planting decreased fruit firmness of the beefsteak tomato ‘Skyway’ (Figure 1C) by 42.4% relative to Spring planting (Figure 1D).

**TABLE 5:** Fruit firmness (kgf) of fruit from grafted beefsteak tomato ‘Skyway’ and grape tomato ‘BHN 1022’ in Spring and Fall plantings.

Rootstock	Scion		Planting season	
	BHN 1022	Skyway	Spring	Fall
DR0141TX	2.84 ± 0.17*Aa*	2.24 ± 0.17*Bd*	2.63 ± 0.17*Ab*	2.45 ± 0.17*Aa*
Estamino	2.82 ± 0.17*Aa*	2.31 ± 0.17*Bcd*	2.69 ± 0.17*Ab*	2.44 ± 0.17*Aa*
RST-04-106-T	2.93 ± 0.17*Aa*	3.20 ± 0.18*Aa*	3.57 ± 0.17*Aa*	2.56 ± 0.18*Ba*
Shield	3.09 ± 0.17*Aa*	2.71 ± 0.17*Abc*	3.39 ± 0.17*Aa*	2.41 ± 0.17*Ba*
Non-grafted	3.15 ± 0.17*Aa*	2.82 ± 0.17*Aab*	3.27 ± 0.17*Aa*	2.69 ± 0.17*Ba*

DR0141TX, tomato scions grafted onto rootstock ‘DR0141TX’; Estamino, tomato scions grafted onto rootstock ‘Estamino’; RST-04-106-T, tomato scions grafted onto rootstock ‘RST-04-106-T’; Shield, tomato scions grafted onto ‘Shield’; Non-grafted, non-grafted tomato scion controls. Mean ± SE (standard error); Means followed by the same uppercase letter within a row, and means followed by the same lowercase letter within a column are not significantly different at *p* ≤ 0.05 according to Fisher’s LSD test.

Fruit dry matter content was affected by planting season, rootstock, scion, and planting season × scion interactions (Table 1). Vigorous rootstocks ‘DR0141TX’ and ‘Estamino’, and mid-vigor rootstock ‘RST-04-106-T’ consistently decreased fruit dry matter content compared with the least vigorous rootstock ‘Shield’ and the non-grafted controls by 7.7% on average, while the latter two treatments were similar to each other (Table 4). Grape tomato fruit had 10.4% greater dry matter content in Fall planting than in Spring planting, while for beefsteak tomato, there were no seasonal impacts on fruit dry matter content.

### Soluble solids content, titratable acidity, and pH

Fruit SSC, TA, and SSC/TA were affected by planting season, rootstock, and scion, while SSC and TA were also impacted by the interactions between planting season and scion (Table 6). Grafting of both scions with the vigorous rootstocks (‘DR0141TX’ and ‘Estamino’) and mid-vigor rootstock (‘RST-04-106-T’) decreased fruit SSC relative to the non-grafted controls by 10.0% on average (Table 7). Among rootstocks, fruit from grafted ‘DR0141TX’ and ‘Estamino’ plants had 10.6% lower SSC relative to those of ‘Shield’, while fruit SSC with ‘RST-04-106-T’ was greater than with ‘Estamino’. Fruit SSC of ‘DR0141TX’ did not differ from ‘Estamino’ and ‘RST-04-106-T’. Only grafting with ‘Estamino’ decreased fruit TA in both scions relative to the non-grafted controls (Table 7). In terms of SSC/TA, grafting with ‘DR0141TX’ or ‘Estamino’ decreased SSC/TA of both scions by 6.3% on average relative to the non-grafted controls (Table 8). These two rootstock treatments also resulted in lower SSC/TA compared with that of ‘Shield’.

**TABLE 6:** Analysis of variance of the effects of planting season (Spring and Fall), rootstock (‘DR0141TX’, ‘Estamino’, ‘RST-04-106-T’, ‘Shield’, and the non-grafted scion controls), and scion (grape tomato ‘BHN 1022’ and beefsteak tomato ‘Skyway’) on tomato fruit soluble solids content (SSC), titratable acidity (TA), SSC/TA, pH, total antioxidant capacity, and contents of total carotenoids, lycopene, *β*-carotene, ascorbic acid, and total phenolics.

Effect	SSC	TA	SSC/TA	pH	Total antioxidant capacity	Total carotenoid	Lycopene	*β*-carotene	Ascorbic acid	Total phenolics
P	***	***	*	**	NS	**	NS	NS	*	*
R	***	*	***	NS	**	*	*	NS	***	***
S	***	***	***	***	***	NS	NS	***	***	***
P × R	NS	NS	NS	**	***	NS	NS	NS	***	*
P × S	*	**	NS	**	***	NS	NS	*	NS	NS
S × R	NS	NS	NS	*	NS	NS	NS	NS	NS	*
P × S × R	NS	NS	NS	NS	NS	NS	NS	NS	NS	NS

P = planting season; R = rootstock; S = scion; P × R = planting season × rootstock interaction; P × S = planting season × scion interaction; S × R = scion × rootstock interaction; P × S × R = planting season × scion × rootstock interaction. NS, *, **, *** Non-significant or significant at *p* at 0.05, 0.01, or 0.001, respectively.

**TABLE 7:** Soluble solids content (SSC), titratable acidity (TA), and *β*-carotene content of fruit from grafted beefsteak tomato ‘Skyway’ and grape tomato ‘BHN 1022’ in Spring and Fall plantings.

Treatment	SSC (Brix°)		TA (% citric acid)		*β*-carotene (μg/100 g FW)	
	BHN 1022	Skyway	BHN 1022	Skyway	BHN 1022	Skyway
**Planting season**						
Spring	4.92 ± 0.11 Ab	4.12 ± 0.11 Aa	0.427 ± 0.010 Ab	0.279 ± 0.010 Bb	694.40 ± 55.81 Aa	282.69 ± 55.81 Ba
Fall	5.72 ± 0.11 Aa	4.39 ± 0.12 Ba	0.554 ± 0.010 Aa	0.319 ± 0.010 Ba	516.71 ± 55.81 Ab	394.86 ± 56.47 Aa

**Rootstock**						
DR0141TX	4.60 ± 0.10 cd		0.394 ± 0.009 ab		403.28 ± 45.86	
Estamino	4.38 ± 0.10 d		0.378 ± 0.009 b		439.99 ± 45.86	
RST-04-106-T	4.82 ± 0.10 bc		0.394 ± 0.009 ab		503.49 ± 47.12	
Shield	5.02 ± 0.10 ab		0.394 ± 0.009 ab		501.67 ± 45.86	
Non-grafted	5.11 ± 0.10 a		0.413 ± 0.009 a		512.39 ± 45.86	

DR0141TX, tomato scions grafted onto rootstock ‘DR0141TX’; Estamino, tomato scions grafted onto rootstock ‘Estamino’; RST-04-106-T, tomato scions grafted onto rootstock ‘RST-04-106-T’; Shield, tomato scions grafted onto ‘Shield’; Non-grafted, non-grafted tomato scion controls. Mean ± SE (standard error); Means followed by the same uppercase letter within a row, and means followed by the same lowercase letter within a column are not significantly different at *p* ≤ 0.05 according to Fisher’s LSD test.

**TABLE 8:** Soluble solids content (SSC) to titratable acidity (TA) ratio and total carotenoid and lycopene contents of fruit from grafted beefsteak tomato ‘Skyway’ and grape tomato ‘BHN 1022’ in Spring and Fall plantings.

Treatment	SSC/TA	Total carotenoids (mg *β*CE/100 g FW)	Lycopene (μg/100 g FW)
**Planting season**			
Spring	13.13 ± 0.28a	20.24 ± 1.05b	1640.32 ± 144.81
Fall	12.11 ± 0.28b	25.45 ± 1.07a	1474.67 ± 145.38

**Scion**			
BHN 1022	10.95 ± 0.23b	23.92 ± 1.05	1475.60 ± 113.50
Skyway	14.29 ± 0.23a	21.77 ± 1.07	1639.39 ± 114.23

**Rootstock**			
DR0141TX	12.17 ± 0.28*bc*	21.21 ± 1.33*bc*	1417.94 ± 137.36*bc*
Estamino	11.97 ± 0.28c	20.10 ± 1.33c	1302.87 ± 137.36c
RST-04-106-T	12.77 ± 0.29*ab*	24.88 ± 1.38a	1630.32 ± 141.11*ab*
Shield	13.29 ± 0.28a	24.22 ± 1.33*ab*	1702.84 ± 137.36*ab*
Non-grafted	12.88 ± 0.28a	23.82 ± 1.33*ab*	1733.49 ± 137.36a

*β*CE, *β*-carotene equivalent. DR0141TX, tomato scions grafted onto rootstock ‘DR0141TX’; Estamino, tomato scions grafted onto rootstock ‘Estamino’; RST-04-106-T, tomato scions grafted onto rootstock ‘RST-04-106-T’; Shield, tomato scions grafted onto ‘Shield’; Non-grafted, non-grafted tomato scion controls. Mean ± SE (standard error); Means followed by the same letter are not significantly different at *p* ≤ 0.05 according to Fisher’s LSD test.

For the grape tomato ‘BHN 1022’, SSC of fruit from the Fall planting was 16.3% greater than from the Spring planting, while for the beefsteak tomato ‘Skyway’, no seasonal effects were detected (Table 7). In Fall planting, SSC of ‘BHN 1022’ was greater than ‘Skyway’, which was not observed in Spring planting. For both scions, fruit TA was higher in Fall planting than in Spring planting, with the differences being greater for ‘BHN 1022’. Moreover, fruit TA of ‘BHN 1022’ was greater than ‘Skyway’ in both seasons with the difference being more pronounced in Fall planting. The ‘Skyway’ fruit had a 30.5% higher SSC/TA than ‘BHN 1022’ fruit (Table 8). Spring planting also resulted in 8.4% greater SSC/TA in tomato fruit relative to Fall planting.

**FIGURE 2:**
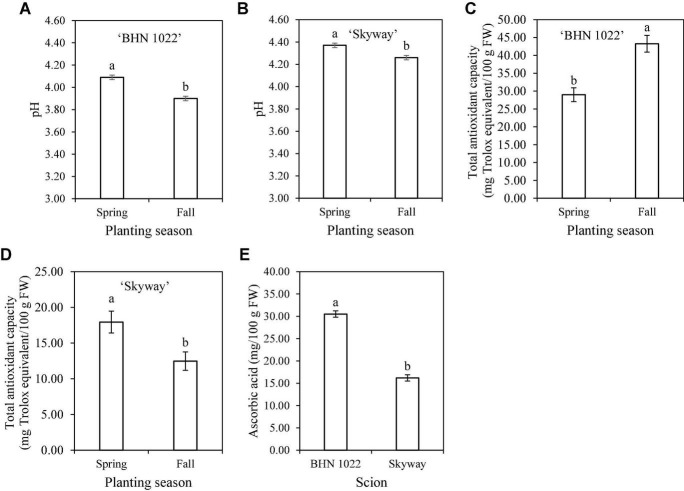
Tomato fruit pH, total antioxidant capacity, and ascorbic acid content as affected by scion or scion × planting season interaction. **(A)** pH of ‘BHN 1022’ grape tomato fruit. **(B)** pH of ‘Skyway’ beefsteak tomato fruit. **(C)** Total antioxidant capacity of ‘BHN 1022’ fruit. **(D)** Total antioxidant capacity of ‘Skyway’ fruit. **(E)** Ascorbic acid content of ‘BHN 1022’ and ‘Skyway’ fruit. Error bars represent standard errors. Means followed by the same letter are not significantly different at *p* ≤ 0.05 according to Fisher’s LSD test.

Fruit pH was affected by planting season, scion, and the two-way interactions among planting season, rootstock, and scion (Table 6). For the grape tomato scion, ‘DR0141TX’ and ‘Estamino’, the two vigorous rootstocks increased fruit pH relative to the non-grafted control, while ‘RST-04-106-T’ and ‘Shield’ were similar to all treatments (Table 9). The pH values of beefsteak tomato were similar among all treatments. In addition, when grafted with the same rootstocks or non-grafted, fruit of ‘Skyway’ showed higher fruit pH than that of ‘BHN 1022’ while the extent was dependent on treatments. In Spring planting, all rootstocks treatments resulted in fruit with similar pH values regardless of scion cultivar; in Fall planting, ‘DR0141TX’, ‘Estamino’, and ‘RST-04-106-T’ increased fruit pH relative to the non-grafted controls. Fruit of plants in Spring planting had greater pH than those in Fall planting irrespective of rootstock–scion combinations, although the extent varied with treatments. Furthermore, for both scions, fruit pH was higher in the Spring than in the Fall, with the seasonal differences being greater for ‘BHN 1022’ than ‘Skyway’ (Figures 2A,B).

**TABLE 9:** Fruit pH and total phenolic content of fruit from grafted beefsteak tomato ‘Skyway’ and grape tomato ‘BHN 1022’ in Spring and Fall plantings.

Rootstock	pH				Total phenolics (mg GAE/100 g FW)			
	Scion		Planting season		Scion		Planting season	
	BHN 1022	Skyway	Spring	Fall	BHN 1022	Skyway	Spring	Fall
DR0141TX	4.01 ± 0.02*Ba*	4.32 ± 0.02*Aa*	4.25 ± 0.02*Aa*	4.09 ± 0.02*Ba*	34.79 ± 1.00*Ab*	21.12 ± 1.00*Bbc*	29.95 ± 1.03*Ac*	25.96 ± 1.03*Ba*
Estamino	4.01 ± 0.02*Ba*	4.30 ± 0.02*Aa*	4.21 ± 0.02*Aa*	4.10 ± 0.02*Ba*	35.51 ± 1.00*Ab*	19.52 ± 1.00*Bc*	28.21 ± 1.03*Ac*	26.81 ± 1.03*Aa*
RST-04-106-T	3.99 ± 0.02*Bab*	4.33 ± 0.02*Aa*	4.22 ± 0.02*Aa*	4.10 ± 0.02*Ba*	35.57 ± 1.00*Ab*	24.04 ± 1.06*Ba*	32.29 ± 1.03*Ab*	27.32 ± 1.09*Ba*
Shield	3.99 ± 0.02*Bab*	4.32 ± 0.02*Aa*	4.23 ± 0.02*Aa*	4.07 ± 0.02*Bab*	40.12 ± 1.06*Aa*	23.52 ± 1.00*Ba*	34.85 ± 1.03*Aa*	28.79 ± 1.09*Ba*
Non-grafted	3.96 ± 0.02*Bb*	4.31 ± 0.02*Aa*	4.23 ± 0.02*Aa*	4.05 ± 0.02*Bb*	38.17 ± 1.00*Aa*	23.29 ± 1.00*Bab*	33.46 ± 1.03*Aab*	28.00 ± 1.03*Ba*

GAE, gallic acid equivalent. DR0141TX, tomato scions grafted onto rootstock ‘DR0141TX’; Estamino, tomato scions grafted onto rootstock ‘Estamino’; RST-04-106-T, tomato scions grafted onto rootstock ‘RST-04-106-T’; Shield, tomato scions grafted onto ‘Shield’; Non-grafted, non-grafted tomato scion controls. Mean ± SE (standard error); Means followed by the same uppercase letter within a row, and means followed by the same lowercase letter within a column are not significantly different at *p* ≤ 0.05 according to Fisher’s LSD test.

### Total antioxidant capacity and contents of antioxidant compounds

Fruit total antioxidant capacity was affected by rootstock, scion, and planting season × rootstock interactions, as well as planting season × scion interactions (Table 6). In Spring planting, grafting onto the two vigorous rootstocks (‘DR0141TX’ and ‘Estamino’) decreased fruit total antioxidant capacity of both scion cultivars by 18.8% on average relative to the non-grafted controls, while grafting with ‘Shield’ or ‘RST-04-106-T’ did not exhibit any significant impact (Table 10). However, different rootstock impacts were observed in Fall planting, with fruit of plants grafted onto ‘DR0141TX’ or ‘Shield’ resulting in 15.8% lower total antioxidant capacity on average than fruit on the non-grafted controls. Among rootstocks, ‘RST-04-106-T’ had greater total antioxidant capacity than ‘Shield’. Seasonal variations of rootstock impacts on total antioxidant capacity were only observed for ‘Estamino’, with the Spring planting being lower than the Fall planting. Fruit of the grape tomato ‘BHN 1022’ from the Spring planting had a 33.0% lower total antioxidant capacity than fruit from the Fall planting (Figure 2C). In contrast, fruit of the beefsteak tomato ‘Skyway’ in Spring planting had a 43.9% higher total antioxidant capacity than in Fall planting (Figure 2D).

**TABLE 10:** Total antioxidant capacity and ascorbic acid content of fruit from grafted beefsteak tomato ‘Skyway’ and grape tomato ‘BHN 1022’ in Spring and Fall plantings.

Rootstock	Total antioxidant capacity (mg Trolox equivalent/100 g FW)		Ascorbic acid (mg/100 g FW)	
	Spring	Fall	Spring	Fall
DR0141TX	19.87 ± 1.73*Ac*	23.69 ± 1.88*Abc*	22.77 ± 0.90*Ac*	20.33 ± 0.90*Ac*
Estamino	19.96 ± 1.73*Bc*	26.35 ± 1.98*Aabc*	23.67 ± 0.90*Ac*	20.83 ± 0.90*Abc*
RST-04-106-T	23.95 ± 1.89*Ab*	27.16 ± 2.08*Aab*	25.97 ± 0.90*Ab*	21.35 ± 0.91*Babc*
Shield	27.82 ± 2.04*Aa*	22.97 ± 1.86*Ac*	27.57 ± 0.90*Aa*	21.68 ± 0.90*Bab*
Non-grafted	24.54 ± 1.91*Aab*	27.72 ± 2.03*Aa*	27.03 ± 0.90*Aab*	22.36 ± 0.90*Ba*

DR0141TX, tomato scions grafted onto rootstock ‘DR0141TX’; Estamino, tomato scions grafted onto rootstock ‘Estamino’; RST-04-106-T, tomato scions grafted onto rootstock ‘RST-04-106-T’; Shield, tomato scions grafted onto ‘Shield’; Non-grafted, non-grafted tomato scion controls. Mean ± SE (standard error); Means followed by the same uppercase letter within a row, and means followed by the same lowercase letter within a column are not significantly different at *p* ≤ 0.05 according to Fisher’s LSD test.

Fruit total carotenoid content was only affected by planting season and rootstock (Table 6). The total carotenoid content of fruit from both scions grafted with the vigorous rootstock ‘Estamino’ was 15.6% lower than the non-grafted controls, while no differences were found among the non-grafted controls and other rootstock treatments (Table 8). In addition, grafting with the mid-vigor rootstock ‘RST-04-106-T’ resulted in greater total carotenoid content than ‘DR0141TX’ and ‘Estamino’ treatments, and fruit from ‘Shield’-grafted plants had higher total carotenoid content than ‘Estamino’ as well. Total carotenoid contents were found to be 25.7% greater in Fall planting than in Spring planting. Fruit lycopene content was only affected by rootstock (Table 6), with ‘DR0141TX’ and ‘Estamino’, the two vigorous rootstocks, causing fruit to have 18.2% lower lycopene content than the non-grafted controls (Table 8). The content of *β*-carotene was affected by scion and planting season × scion interactions, but not rootstock (Table 6). For grape tomato ‘BHN 1022’, Spring planting increased fruit *β*-carotene content relative to Fall planting by 34.4%; however, planting season had no effects on *β*-carotene content in the beefsteak tomato (Table 7). ‘BHN 1022’ also had greater *β*-carotene contents than ‘Skyway’ in Spring planting, but this difference disappeared in Fall planting.

Planting season, rootstock, scion, and planting season × rootstock interactions affected ascorbic acid content in tomato fruit (Table 6). In Spring planting, ‘DR0141TX’ and ‘Estamino’ decreased fruit ascorbic acid content by 14.1% on average in contrast to the non-grafted controls (Table 10). Moreover, among rootstocks, fruit from scions grafted with ‘Shield’ had the highest ascorbic acid content, followed by those of ‘RST-04-106-T’ and then by those of ‘DR0141TX’ and ‘Estamino’. In Fall planting, fruit from scions grafted with ‘DR0141TX’ or ‘Estamino’ had 8.0% lower ascorbic acid contents than the non-grafted control fruit, while ‘RST-04-106-T’ and ‘Shield’ fruits were similar to the non-grafted controls (Table 10). When grafted with ‘RST-04-106-T’ or ‘Shield’ or non-grafted, fruit from the Spring planting had greater ascorbic acid content than those in Fall planting. Moreover, ‘BHN 1022’ fruit contained 88.3% more ascorbic acid content than ‘Skyway’ regardless of rootstock and planting season (Figure 2E).

Total phenolic content of fruit was affected by planting season, rootstock, and scion, as well as planting season × rootstock and scion × rootstock interactions (Table 6). For grape tomato ‘BHN 1022’, grafting with ‘DR0141TX’, ‘Estamino’, or ‘RST-04-106-T’ decreased fruit total phenolic content by 7.5% compared with the non-grafted control, but the latter did not differ from ‘Shield’ (Table 9). In addition, ‘BHN 1022’ fruit produced on ‘Shield’ had higher total phenolic content than on all other rootstock treatments. For beefsteak tomato ‘Skyway’, fruit on plants grafted with ‘Estamino’ had 16.2% lower total phenolic content than the fruit of the non-grafted control, while the fruit on other rootstocks were similar to the non-grafted control. ‘Shield’ and ‘RST-04-106-T’ rootstocks also led to greater total phenolic contents compared with ‘DR0141TX’ and ‘Estamino’ rootstocks. ‘BHN 1022’ fruit had greater fruit total phenolic content than ‘Skyway’ fruit, while the extent was treatment-dependent. For the Spring planting, ‘DR0141TX’ and ‘Estamino’ decreased fruit total phenolic contents for both scions by 13.1% on average relative to the non-grafted controls, while total phenolic content was similar among the non-grafted controls and the other two rootstock treatments. Rootstock comparisons followed the pattern of ‘Shield’ > ‘RST-04-106-T’ > ‘DR0141TX’ and ‘Estamino’. For the Fall planting, rootstocks did not show any impact on fruit total phenolic content. For all treatments, except ‘Estamino’, Spring planting resulted in higher total phenolic content than Fall planting with varying degrees.

### Characterizing fruit quality profiles in relation to planting season, scion, and rootstock

**FIGURE 3:**
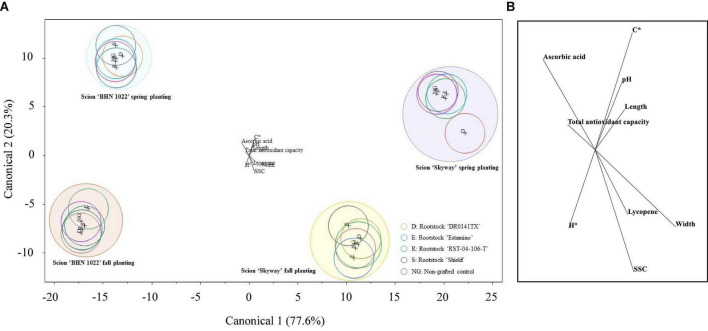
Plot of the first and second canonical functions of canonical discriminant analysis with the forward stepwise variable selection method based on *p* ≤ 0.05 for different planting season-scion-rootstock treatments using tomato fruit physical and chemical attributes. The circle represents the 95% confidence interval. **(A)** Canonical plot. **(B)** Fruit quality parameters contributing to treatment separation.

Canonical discriminant analysis of all the fruit quality attributes measured for all the treatments was conducted to examine the multivariate separation pattern of fruit quality attributes among planting season, scion, and rootstock treatments. The canonical plot showed that the cumulative percentage of the variance explained by the first two canonical variables was 97.89% of the total sample variance (Figure 3A). Fruit length, width, C*, *H*°, SSC, pH, total antioxidant capacity, lycopene, and ascorbic acid contents contributed to differentiating the treatments (Figure 3B). Scion and planting season combinations were clearly differentiated, but there was a lack of separation between rootstock treatments and non-grafted scions. Regardless of planting season, grape tomato ‘BHN 1022’ was separated from beefsteak tomato ‘Skyway’ by having relatively greater total antioxidant capacity and ascorbic acid content in addition to its smaller size. Irrespective of scion cultivar, Spring planting differed from Fall planting by having greater fruit C* and pH. The only separation of rootstock treatments was observed in ‘Skyway’ in Spring planting, indicating that grafting with ‘DR0141TX’ tended to lower ascorbic acid content compared with the other treatments.

## Discussion

### Effects of rootstock on tomato fruit quality

The hue of fruit from tomato plants grafted with the most vigorous rootstock was more orange than red compared with those fruit from less vigorous rootstock treatments and the non-grafted controls, suggesting rootstock vigor might adversely impact fruit color tone. There was no clear pattern of rootstock vigor impacts on fruit C* considering scion cultivars and planting seasons, indicating the purity of fruit skin color was largely affected by rootstock–scion combinations and environmental conditions. More research is needed regarding rootstock vigor impacts on tomato fruit color development and fruit ripening.

Rootstock vigor did not have consistent effects on fruit length, but the fruit width was increased when grafted with vigorous rootstocks or a rootstock of medium-vigor regardless of scion cultivar and planting season. Negative impacts of vigorous rootstocks on fruit firmness were only observed for beefsteak tomato but not grape tomato and only in Spring planting but not in Fall planting. Lang et al. (2020) also evaluated the same three rootstocks (‘DR0141TX’, ‘Estamino’, and ‘RST-04-106-T’) in a 2-year high tunnel study and found that they did not impact beefsteak tomato firmness, which was in agreement with our findings in Fall planting. Our previous study involving both ‘DR0141TX’ and ‘Estamino’ showed that the impacts of these two rootstocks on grape tomato firmness varied with harvest dates (Gong et al., 2022). In terms of effects of rootstock genetic background on tomato fruit firmness, we found that interspecific rootstock ‘Estamino’ consistently decreased fruit firmness of beefsteak tomato but not grape tomato in both planting seasons, while the intraspecific rootstock ‘Shield’ showed no impacts. However, Khah et al. (2006) and Buller et al. (2013) did not observe any effect of interspecific rootstocks on beefsteak tomato fruit firmness in their studies. The differences in beefsteak tomato firmness in this study compared with those other studies and the differences in fruit firmness of the two tomato scion types when grafted with the same rootstock highlight the need to examine fruit firmness with specific rootstock–scion combinations. As phytohormone cues, epigenetic regulations, and transcriptional modulations of cell wall structure–related genes are involved in the process of tomato fruit ripening and texture change (Wang and Seymour, 2022), different rootstock–scion combinations may modify these processes differently, leading to differences in fruit firmness.

Rootstock vigor tended to negatively affect fruit dry matter content regardless of scion and planting season, while the decrease did not strictly follow the level of rootstock vigor. Krumbein and Schwarz (2013) found that rootstock impacts on fruit dry matter content were affected by rootstock/scion combinations in ways that may not have been necessarily related to rootstock vigor, which is partially in line with our findings. However, our results differed from Mauro et al. (2020) who reported that rootstocks, irrespective of their vigor, did not show any effects on fruit dry matter content. Consistently lower tomato fruit dry matter content with grafting onto interspecific rootstocks (‘Estamino’) in the present study was also observed by Turhan et al. (2011) and Djidonou et al. (2016). Moreover, our previous studies involving three vigorous rootstocks (‘DR0141TX’, ‘Estamino’, and ‘Multifort’) showed that rootstock impacts on fruit dry matter content of grape tomato fruit varied with harvest dates and scion cultivars (Gong et al., 2022). In contrast, Gioia et al. (2010) demonstrated that interspecific rootstocks (*S. lycopersicum* × *S. habrochaites*) did not affect fruit dry matter content regardless of harvest dates. Guichard et al. (2001) suggested that fruit dry matter content was associated with a balance between water and assimilate influx into fruit (i.e., between the influx from phloem and xylem). It has been shown that phloem flow into fruit is relatively insensitive to plant water status, while xylem transportation is closely related to plant water status (Hou et al., 2020). Rootstock may affect tomato fruit dry matter content by adjusting plant water status.

In this study, rootstock effects on tomato fruit SSC, TA, and SSC/TA did not strictly follow their vigor, which is in line with Krumbein and Schwarz (2013) and Mauro et al. (2020). Nevertheless, our findings showed that the least vigorous rootstock tested had no impact on these quality attributes, whereas the most vigorous rootstock could lead to reduced levels, especially in SSC. In a greenhouse study, Turhan et al. (2011) reported that grafting with interspecific rootstocks (*S. lycopersicum* × *S. habrochaites*) consistently decreased tomato fruit SSC, which is consistent with our findings on ‘Estamino’ for both scions. However, they reported that these rootstocks increased TA relative to the non-grafted controls, while fruit pH was not affected for three large-fruited tomato scions, which differs from our observations. Djidonou et al. (2016, 2017) found that grafting beefsteak tomato with either of two vigorous interspecific rootstocks (*S. lycopersicum* × *S. habrochaites*) resulted in similar fruit SSC, TA, SSC/TA, and pH as the non-grafted control in both open field (Djidonou et al., 2016) and greenhouse conditions (Djidonou et al., 2017). Gioia et al. (2010) also reported an absence of interspecific rootstock (*S. lycopersicum* × *S. habrochaites*) effects on beefsteak tomato fruit SSC, TA, and SSC/TA in greenhouse production, but they observed significant differences in these attributes between harvesting dates. For cherry tomato, grafting with interspecific rootstocks [(*S. lycopersicum* × *S. habrochaites*) or (*S. lycopersicum* × *S. pimpinellifolium*)] also did not affect fruit SSC, TA, and pH in greenhouse condition (Parisi et al., 2022). These mixed results indicate that rootstocks with similar genetic backgrounds could exert different impacts on tomato fruit sensory attributes depending on the scion genotype and growing conditions. Our previous findings with ‘DR0141TX’ and ‘Estamino’ showed that rootstock effects on grape tomato fruit SSC, TA, SSC/TA, and pH were scion cultivar and harvest date dependent (Gong et al., 2022). According to Lang et al. (2020), the effects of ‘DR0141TX’, ‘Estamino’, and ‘RST-04-106-T’ on beefsteak tomato SSC, TA, and SSC/TA varied with years, whereas pH values were not affected by these rootstocks. In our study, the rootstock impact on fruit pH was only seen in the grape tomato and Fall planting. Another study by Lang and Nair (2019) involving ‘RST-04-106-T’ rootstock found the year × rootstock interaction effect on fruit SSC. These results highlight the effects of environmental factors on grafted tomato fruit sensory quality attributes.

The variations in SSC as affected by rootstocks seemed to be associated with the variations of fruit dry matter content, as tomato fruit SSC and dry matter content are highly correlated (Carli et al., 2011), and variations in tomato fruit dry matter content are primarily due to SSC (Young et al., 1993). However, a statistically significant decrease in dry matter content may not result in a similar extent of reduction in SSC that is also statistically significant (Djidonou et al., 2016) and vice versa (Mauro et al., 2020).

In this study, lower fruit lycopene contents observed from plants grafted onto the two vigorous rootstocks ‘DR0141TX’ or ‘Estamino’ corresponded to larger *H*° of the fruit surface. However, rootstock effects on fruit lycopene, *β*-carotene, total carotenoids, and total phenolic contents did not strictly follow rootstock vigor. Interestingly, fruit total antioxidant capacity and ascorbic acid content decreased as rootstock vigor increased in Spring planting, but this was not observed in Fall planting. According to Mauro et al. (2020), tomato fruit lycopene content decreased with increased rootstock vigor, but tomato ascorbic acid and *β*-carotene contents did not respond to rootstock vigor. Krumbein and Schwarz (2013) also reported that tomato fruit *β*-carotene content did not vary based on rootstock vigor, whereas fruit lycopene and total carotenoid contents varied with rootstock cultivar and light intensity. More research is needed to fully characterize rootstock vigor and its impact on fruit compositional quality. In our study, ‘Estamino’ consistently decreased the contents of fruit lycopene, total carotenoids, and ascorbic acid compared with the non-grafted controls irrespective of scion and planting season. In contrast, Parisi et al. (2022) found that grafting a small-sized tomato (around 32 g/fruit) with the interspecific rootstock [(*S. lycopersicum* × *S. habrochaites*) or (*S. lycopersicum* × *S. pimpinellifolium*)] resulted in similar ascorbic acid content but consistently lower *β*-carotene content compared with the non-grafted control, while fruit lycopene content varied with rootstock cultivar. Djidonou et al. (2016, 2017) found that grafting with the vigorous interspecific rootstocks ‘Beaufort’ or ‘Multifort’ did not affect beefsteak tomato fruit lycopene and *β*-carotene contents relative to the non-grafted controls in both open-field (Djidonou et al., 2016) and greenhouse conditions (Djidonou et al., 2017). However, in the open-field condition, these rootstocks resulted in lower fruit ascorbic acid content (Djidonou et al., 2016) whereas, in the greenhouse, rootstock effects on tomato fruit ascorbic acid content varied with harvest dates (Djidonou et al., 2017).

It is very likely that ‘DR0141TX’ is also an interspecific rootstock based on our previous phenotyping studies by growing non-grafted rootstock plants (data not shown). Thus, it is not surprising to observe similar effects of ‘DR0141TX’ and ‘Estamino’ on tomato fruit quality. More information about the genetic background of different rootstocks would help unraveling the rootstock effects on tomato fruit quality.

Because rootstock × planting season interactions were detected for fruit firmness, pH, total antioxidant capacity, ascorbic acid, and total phenolic contents, more research is warranted regarding seasonal effects on rootstock performance in order to select suitable rootstock for different production seasons. In general, more vigorous rootstocks showed greater negative impacts on tomato fruit quality in our study; however, it is unclear how this impact might be linked to grafting transmissible small molecules (Zhou et al., 2022) and grafting-induced changes in gene expression as well as epigenetic changes in the scion (Colling, 2022). Future studies of genes related to phenotypic vigor and their effects on fruit quality could help elucidate rootstock vigor-induced tomato quality variations.

Vegetative (‘DR0141TX’) and generative (‘Estamino’) rootstocks showed similar impacts on all of the fruit quality attributes measured in this high tunnel tomato production study. Future research investigating different growing systems with different environmental conditions is needed to better understand the potential alteration of fruit quality, if any, as related to rootstock trait characterization.

### Effects of planting season and scion on tomato fruit quality

Planting season × scion interactions were detected for most fruit physical parameters (Table 1). The tomatoes from the Spring planting tended to be brighter and redder than those from the Fall planting for both scions. The color of a ripe red tomato is largely determined by the ratio of two pigments lycopene and *β*-carotene (Setyorini, 2021), and the red color of the fruit originates from lycopene. The redder color of fruit in Spring planting could be due to a higher lycopene to *β*-carotene ratio in the fruit skin. However, fruit lycopene content did not vary by planting season nor did *β*-carotene content vary consistently based on planting season for both scions. It is noteworthy that the extent to which the grape tomato scion ‘BHN 1022’ and the beefsteak tomato scion ‘Skyway’ differed in their responses to planting seasons was manifested by the magnitude of the fruit color changes.

When examining the fruit size and dry matter content variations of these two scions over planting seasons, it was interesting to find that grape tomato ‘BHN 1022’ showed a moderate increase in fruit length as well as dry matter content in Fall planting. However, beefsteak tomato ‘Skyway’ produced much smaller fruit in Fall planting regardless of grafting status but comparable fruit dry matter content as in Spring planting. These results indicate that ‘BHN 1022’ and ‘Skyway’ are likely to be differently affected by planting seasons. The fruit size increase in ‘BHN 1022’ observed in Fall planting relative to the Spring planting was surprising as the whole-season average marketable fruit weight decreased from 10.4 g in the Spring to 9.4 g in the Fall (Gong, 2022). This discrepancy could be due to the differences in sample sizes.

A significant decrease in fruit size of beefsteak tomato ‘Skyway’ in Fall planting relative to the Spring planting regardless of rootstock is well in line with the lower whole-season average marketable fruit weight (from 314 to 172 g/fruit) (Gong, 2022). Cell division occurs in the first 2 weeks after tomato fruit set, which determines fruit growth potential; after that, cell elongation lasts around 3–5 weeks, leading to a rapid expansion of tomato fruit (Van de Wal, 2017). Source/sink balance within the plant (Guichard et al., 2001) and water availability have been suggested to affect tomato fruit size (Atherton and Rudich, 2012). During the fruit set and development stage of tomato, tomato plants are source-limited (Li et al., 2015), and the low temperature and light intensity in Fall planting probably could not meet the fruit demand of photosynthate, leading to fruit size reduction in Fall planting. In addition, under low temperature, the viscosity of soil water increased while root hydraulic conductance decreased regardless of rootstock used (Bristow et al., 2021), leading to mild water stress, and potentially lower water supply to fruit. However, the dry matter content of ‘Skyway’ in Fall planting was comparable to that in Spring planting, suggesting negligible water stress. Interestingly, a significant decrease in fruit size in Fall planting was not observed in ‘BHN 1022’. During the fruit set and development stage of tomato, the extent of source limitation is positively correlated with its potential fruit size (Li et al., 2015), suggesting that large-fruited tomato cultivar maybe be more prone to or experience more severe source limitation compared with small-fruited tomato cultivar, which is in line with our observations.

Interactions between planting season and scion affected fruit SSC and TA. Interestingly, seasonal effects on the scion’s fruit SSC showed a similar pattern as for fruit dry matter content. The Fall planting consistently increased fruit TA relative to the Spring planting although to a different degree for the two scion cultivars. This was because the whole-season temperature was 4°C higher in Spring planting than in Fall planting and higher temperatures during fruit development have been shown to decrease fruit TA as well as malate and citrate concentrations in many crops (Etienne et al., 2013).

In tomato, light intensity and quality, air- and fruit canopy temperature, CO_2_ concentration, and growing system (irrigation, fertilization, grafting, etc.) have been demonstrated to significantly affect fruit carotenoid concentration. Tomato fruit subjected to high irradiance and high temperature showed enhanced carotenoids synthesis, probably because of its role against the resulting oxidative stress, and/or its capacity of dissipating excess absorbed energy in the xanthophyll cycle (Cocaliadis et al., 2014; Ilahy et al., 2019). However, in this study, the total carotenoid content in the warmer, sunnier Spring planting was lower than in Fall planting, suggesting that some factor other than light may explain the higher carotenoid content as was observed in fruit from the Fall planting.

In this study, air temperatures during plant growth and fruit development differed substantially between the two different planting seasons, leading to different fruit harvest (sampling) times for quality assessment relative to transplanting dates. Given the important role that temperature plays in fruit set, size increase, and ripening (Verkerk, 1955; Abdalla and Verkerk, 1968; Adams et al., 2001), it is not surprising to see the seasonal variations of some fruit quality parameters measured in this study. Actually, rootstock impacts on fruit quality were relatively moderate compared with planting season as the canonical discriminant analysis revealed. Our results partially explain why in more than 80% of studies evaluating pH, TA, SSC (74%), and firmness of fruit in tomato grafting research, rootstock had no impacts (Grieneisen et al., 2018). Growing conditions and production systems outweighing rootstock effects on fruit quality have also been observed by Djidonou et al. (2016) and Joshi et al. (2021).

## Conclusion

In this study, rootstock effects on fruit quality were examined when grape tomato ‘BHN 1022’ and beefsteak tomato ‘Skyway’ were grafted with rootstocks of different characteristics and grown in Spring and Fall planting seasons. ‘DR0141TX’ and ‘Estamino’, the two most vigorous rootstocks in this study, showed negative impacts on fruit color, dry matter content, SSC, SSC/TA, and lycopene and ascorbic acid contents for both scions in both planting seasons; the medium vigor rootstock, ‘RST-04-106-T’, decreased fruit dry matter content and SSC; and the least vigorous rootstock, ‘SHIELD RZ F1 (61–802)’, resulted in fruit physical and chemical attributes that were comparable to the non-grafted control regardless of planting season, except for lower total antioxidant capacity in the Fall. Meanwhile, scion cultivar and planting season showed much more pronounced impacts on fruit quality attributes than rootstocks. The fruit color (C* and *H*°), length and width, SSC, pH, total antioxidant capacity, ascorbic acid, and lycopene contents were the main attributes distinguishing different scion-planting season groups.

Rootstocks have been primarily used in greenhouses and grafted with indeterminate tomato scions in long growing cycles. Our research conducted in high tunnels in north central Florida across different seasons provides fundamental information for future in-depth evaluation of rootstock effects on quality properties of determinate tomato cultivars in subtropical conditions under protected culture. In future studies, more rootstock–scion combinations should still be tested in terms of fruit yield and quality across different growing seasons and production systems to provide up-to-date research information on cost-effective use of grafting technology to serve sustainable tomato production. In addition to fruit compositional analysis, consumer sensory evaluation, as well as fruit volatile compound examination, needs to be conducted to better understand rootstock effects on tomato flavor and whether rootstock impacts on quality factors raise practical concerns. In this study carried out in north central Florida, “vegetative” and “generative” rootstocks showed similar impacts on tomato fruit quality under high tunnel organic tomato production in a soil-based system without intensive pruning; however, it is unclear if similar findings would be observed when grafted tomato plants are grown in other types of protected production systems such as soilless culture in typical greenhouses given the major differences in cultural management practices, tomato scion cultivars, nutrient and water supply, environmental control, and duration of the crop cycle.

## Data availability statement

The raw data supporting the conclusions of this article will be made available by the authors, without undue reservation.

## Author contributions

TG designed and performed the experiments, analyzed the data, and drafted the manuscript. XZ and JB supervised TG to design and conduct the experiments and reviewed and edited the manuscript. SH and KK offered advice on research information synthesis and helped revise the manuscript. All authors contributed to the article and approved the submitted version.
